# Comparative evaluation of nano-filled and conventional adhesives for bonding of molar tubes 

**DOI:** 10.6026/97320630017492

**Published:** 2021-04-30

**Authors:** Nishtha Arora, Mohammed Al-jearah

**Affiliations:** 1 MDS Orthodontics & Dentofacial Orthopedics, JCD Dental College, Sirsa, Haryana, India; 2 Orthodontist, Department of Preventive Dental Sciences, Faculty of Dentistry, Najran University, KSA

**Keywords:** Adhesives, molar tubes, Shear bond strength, Adhesive Remnant Index

## Abstract

It is of interest to compare the bonding characteristics of the two nano filled adhesives, Grandio (Voco, Cuxhaven, Germany) and Transbond Supreme LV (TSLV, 3M Unitek, Monrovia, California) with conventional bonding adhesive Transbond XT (TBXT, 3M Unitek) for bonding of molar tubes. 45 extracted human permanent molar teeth, divided into three groups of 15 each, were bonded with stainless steel molar tubes (3M Unitek, USA) using TBXT in Group 1, Grandio in Group 2, TSLV in Group 3. Remnant Index and shear bond strength was evaluated after 24 hrs. of storage with the aid of Instron Universal testing machine and Stereomicroscope respectively. Data were analysed using Analysis of Variance (ANOVA) test, Post-hoc Bonferroni test and Kruskal Wallis test. The mean SBS of Group 1(TBXT) was 13.86±3.27 MPa, Group 2 (Grandio) was 9.48±2.36 MPa and Group 3 (TSLV) was 11.64±2.71 MPa. Both nano-filled adhesives had SBS well above the clinically acceptable range. Assessment of ARI scores and type of bond failure revealed that adhesive failure for TBXT and TSLV and cohesive failure for Grandio. Nano-filled adhesives can be an appropriate substitute for the conventional adhesive for bonding of molar tubes.

## Background:

With the development of bonding technique, the researchers are trying to find the perfect material for bonding that provide appropriate strength to bear the pressure transmitted by brackets through the interaction of archwire/brackets. However, the strength of material should not be so strong that it causes harm to the enamel surface on debonding. In markets, new bonding materials are continuously being introduced. These materials should be approved by tests in a laboratory, then in clinics. For the bonding of orthodontic brackets, the first choice is composite adhesive [1]. The main concern point for orthodontists is the continuation of demineralization around the brackets [2,3]. With the progress in nanotechnology, a number of researchers tried to increase adhesive properties with the help of nanoparticles (Elsharkawy, Callister). Adhesives with nano-technology claim to have a prolonged shelf life, increased stability, improved manipulative advantages, homogeneity, translucency and polishability [6]. Successful bonding of orthodontic attachments highly depends on reliable bonding between attachment and the fixed enamel surface for the entire period of treatment. Any failure of these bonded attachments during the treatment may lead to an increase in treatment duration, material cost, patient discomfort and increased chair side time [7]. Among vast varieties of the adhesive present in markets, Transbond XTTM light cure adhesive (3M Unitek, USA) a conventional orthodontic composite adhesive is acknowledged as a gold standard because of its light-curing property, ideal consistency, great adhesion of tooth/bracket [8,9]. Grandio® a nano-hybrid restorative material (Voco, Cuxhaven, Germany) introduced in 2003 has an incredibly high filler content of 87% w/w, low polymerization shrinkage, amazing surface hardness and good abrasion resistance [10]. Another nano-filled Light Cure adhesive introduced in 2008 with improved shear bond strength as compared to available bonding adhesives is Transbond Supreme LV Low viscosity light cure nanofilled adhesive (3M Unitek, Monrovia, California) [11]. It has been claimed by the manufacturers that it has good strength, wears properties and viscosity that make it an ideal adhesive. Bonded molar tubes (BMTs) have recently emerged as an alternative to molar banding due to improvements in bonding procedures and molar tube design. The advantages of bondable molar tubes are that they reduce chairside time by eliminating time taken for placement of separators, eliminate post‑orthodontic space in between the molars, allowing easier maintenance of oral hygiene, less plaque accumulation and gingival inflammation, thereby reducing the risk of demineralization and caries [12]. Therefore, it is of interest to document data on the comparative evaluation of nano-filled and conventional adhesives for bonding of molar tubes.

## Materials and methods:

### Sample description:

The sample collected for the study consisted of 45 human permanent molars, indicated for extraction, stored at room temperature in 0.9% saline solution (isotonic). The inclusion criteria were: 1. Intact buccal enamel not subjected to any kind of pre-treatment chemicals, 2. No fracture or cracks lines due to extraction forceps, 3. Caries or abrasion¬, 4. Developmental defects.

### Method:

The samples were allocated to 3 groups (15 teeth each) and were embedded in color-coded cold cure acrylic resin blocks up to the junction of cement enamel. The teeth were kept in the centre of the block in which the long axis of the tooth was kept perpendicular to the base of the block. These acrylic blocks were later stored in distilled water at room temperature before bonding.

### Bonding Procedure:

The buccal surface of each mounted tooth was cleaned and polished with pumice slurry and polishing cups using a low-speed handpiece for 10 seconds. After every seven teeth, the polishing cups were changed to obtain a clean bonding surface. Then it was rinsed with water and dried using an oil-free three-way syringe for 20 sec. Each tooth was etched for 30 seconds using N-etch etching gel (Ivoclar Vivadent) containing 37% phosphoric acid and was air-dried until chalky white appearance. Victory seriesTM 0.022" slot MBT prescription upper convertible double molar tubes (3M Unitek, USA) having 12.45 mm2 base area on average were applied for bonding on the buccal surface of the molars. The tubes were handled with bonding tweezers all the time to avoid any contamination of the bonding base.

### The groups were colour coded and the bonded teeth were grouped as:

Group 1 (Red): Transbond XT primer, Transbond XT paste (3M Unitek, USA) and light cured.

Group 2 (Blue): Transbond XT primer, Grandio paste (Voco, Cuxhaven, Germany) and light cured.

Group 3 (Green): Transbond XT primer, Transbond Supreme LV paste (3M Unitek, USA) and light cured.

**Figure 1:**
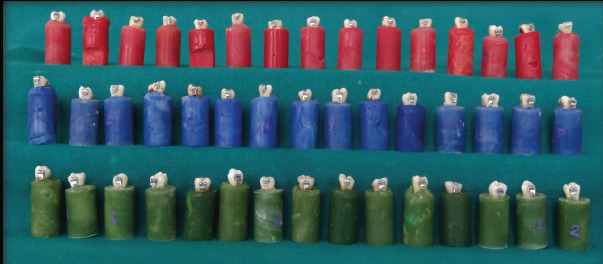
Group 1, Group 2, Group 3 after bonding of molar tubes

The tubes were placed on the buccal surface of each tooth with firm pressure and excess adhesive was removed using a sharp explorer. The tube was kept at 4mm distance from the occlusal surface using a gauge. Then for 40-seconds, positioning towards the light source, the adhesive was light-cured at a distance of 5mm for 10 seconds on each side (mesial, distal, occlusal and gingival). To avoid any variation, a single operator handled all procedures (Figure 1). The specimens after bonding were stored for 24 hours at 37°C in distilled water.

### Testing the strength of the shear bond:

**Figure 2:**
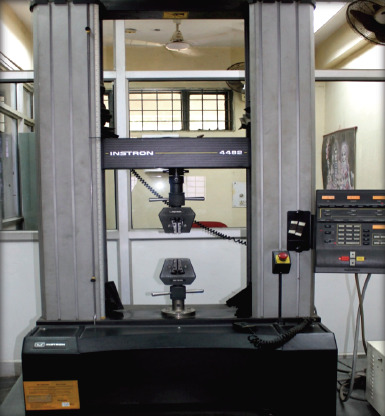
Instron Universal Testing Machine

An occluso-gingival load was applied at the tooth/molar tube interface with standard knife-edge attachment attached to Instron Universal testing machine (Instron 4482, UK) with 100 KN load cell at a crosshead speed of 0.5 mm/min (Figure 2). The force, which produced bond failure, was recorded on the computer. The strength of the shear bond was measured in MPa as follows:

Bond strength calculation:

Bond strength (MPa)= Debonding force values (N) Surface area of molar tube (mm2)

### Scoring Criteria - Adhesive Remnant Index:

After debonding, the teeth were observed with the help of stereomicroscope (SALL 1539, Spectro lab equipment, India) at 20x magnification. Each tooth surface was analysed for the residual composite and the site of bond failure using Adhesive Remnant Index score (ARI) by Bishara and Trulove13 as shown (Table 1 - see PDF).

## Observations and results:

### Strength of shear bond:

The mean strength of Shear bond for Transbond XT (TBXT), Grandio, Transbond Supreme LV (TSLV) were 13.86, 9.48. 11.64 MPa, respectively (Table 2 - see PDF). One way ANOVA test showed highly significant alteration within different groups in Shear bond strength with a p-value < 0.001 (Table 3 - see PDF). Post hoc Bonferroni test showed a highly significant difference in SBS values when TBXT was compared with Grandio. Comparison of Grandio with TSLV and TBXT with TSLV showed a non-significant difference in SBS values (Table 4 - see PDF).

### Scanning Electron Microscope:

Based on a maximum score of Adhesive Remnant Index, one representative molar tube base from each group was selected. Scanning electron micrographs (Hitachi TM 3000, Japan) at a working distance of 40x and 300x and scale bar 150µ and 30µ respectively at 5kV voltage were used to analyse tube surfaces qualitatively.

### Adhesive remnant index:

Group 1 had eight molar tubes (53.3%) showing failure at composite molar tube interface with all the adhesive remaining on the tooth surface. Group 2 had 9 molar tubes (60%) showing failure in the adhesive itself, leaving more than 10% but less than 90% adhesive on the tooth surface. Group 3 had 9 molar tubes (60%) showing failure at composite molar tube interface with more than 90% adhesive remaining on the tooth surface (Table 5 - see PDF). A highly significant relationship was found between groups and ARI scores with the chi-square analysis (p <0.001). Group 1 had mean ARI score1.87 ± 1.13, Group 2 and Group 3 was 3.27 ± 0.88 and 2.27 ± 0.88 respectively (Table 6 - see PDF). The mean difference of ARI frequency distribution for the three groups was highly significant (p<0.001). The Weibull modulus for Group 1 was 6.23, Group 2 and 3 was 4.55 and 4.98 respectively, indicating the greatest bond reliability of Group 1 followed by Group 3 and Group 2 respectively (Table 7- see PDF). The Spearman rank correlation coefficient for Group 1 was 0.701, Group 2 and Group 3 was 0.625 and 0.691 respectively (Table 8 - see PDF). A significant relationship was seen in shear bond strength and ARI scores in all the three groups (p <0.05).

### Scanning Electron Microscopy:

**Figure 5:**
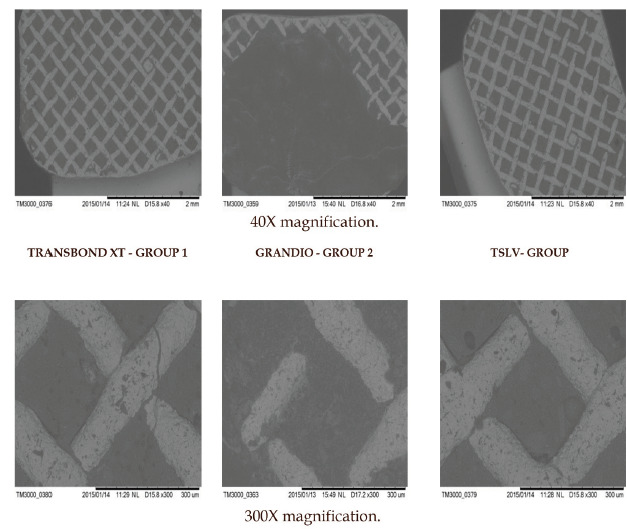
Scanning Electron Micrographs of molar tube bases viewed at 40X and 300X magnification

Scanning electron micrographs of one representative molar tube base from each group, taken at 40x showed a uniform flow of the adhesive in the mesh network. At 300x magnification, greater incidence of air bubbles was observed with Transbond Supreme LV when compared to Transbond XT and Grandio (Figure 5).

## Discussion:

With the continuous development in orthodontics, new materials have introduced for problems with improved quality. The major developments in dentistry were the introduction of acid etches technique by Buonocore (1955) [14] and Newman (1965) [15] was the first to introduce this technique into orthodontics for bonding attachments using epoxy resins. The advent of direct bonding of orthodontic brackets revolutionized the efficacy of clinical practice in orthodontics, both for the patient and the operator. This is important for the successful and efficient orthodontic treatment. The bond strength is very important. Clinically, it is not possible to find the potential of different adhesive materials due to many factors that can affect the longtivity and quality of attachment. However, the best method for the study of the effectiveness of adhesive bonding is an in-vivo test [16]. In-vitro study was performed with the help of mechanical machines provide the best condition for the placement of brackets and good moisture content. With the objective of researching for a superior orthodontic bonding material for molar tubes having reduced polymerization shrinkage, adequate bond strength and improved clinical handling properties, the current in-vitro study was undertaken to compare the nano-filled adhesives like Grandio a nano-hybrid restorative material (Voco, Cuxhaven, Germany) and Transbond Supreme Low Viscosity light cure nanofilled adhesive (3M Unitek, Monrovia, California) with that of traditional orthodontic adhesive, Transbond XT light cure adhesive (3M Unitek). Transbond XT (Group 1) in the present study exhibited shear bond strength of 13.88±3.28 MPa. This is in a similar range as that of the previous studies [6,17], which reported the strength of Transbond XT, is between 5.3 MPa to 20 MPa. The SBS of Grandio (Group 2) in the present study was 9.484 ± 2.37 MPa which was significantly higher than that obtained by previous studies [10,13]. The SBS of Transbond Supreme LV (Group3) was 12.44±2.71MPa, which was similar to results obtained in previous studies [6]. Results of the present study showed that all three groups had strengths well above the clinically acceptable range of 5.9 -7.8 MPa as suggested by Reynolds (1976) [18] and above 7MPa as recommended by Lopez (1980) [19] the maximum bond strength for successful bonding. Intergroup comparison of SBS was significant statistically significant with (p <0.05) between Transbond XT and Grandio. However, the difference between the values of SBS of Transbond XT and Transbond Supreme LV were non-significant (p >0.05). There are two types of bond failure (i) adhesive failure and (ii) cohesive failure. Bond failure between enamel surface and material or between bracket surface and material is called adhesive failure while the bond failure within brackets, within enamel or the material is called cohesive failures. Adhesive Remnant Index is a scale, which is used to measure the percentage of bond failure. In the present study, the predominant mode of bond failure for Transbond XT and Transbond Supreme LV was at adhesive molar tube interface or adhesive in nature leaving adhesive on the surface of a tooth. The predominant mode of failure for Grandio was within the adhesive itself i.e. adhesive present partly on the enamel surface and partly on the bracket base. The intergroup comparison of the ARI among the three groups showed a statistically significant difference (p<0.05). The mean difference of distribution of frequency of the ARI among the three groups was highly significant (p<0.001). The Weibull analysis calculates the probability of fracture as the result of applied load and vice versa. Results of the present study showed the Weibull modulus for Transbond XT was 6.23, Grandio and Transbond Supreme LV was 4.55 and 4.98 respectively, representing the highest bond reliability of Transbond XT followed by Transbond Supreme LV and Grandio respectively. Major bond failure for Grandio was within the adhesive itself i.e. adhesive present partly on the surface of enamel and partly on the bracket base. The intergroup comparison of the ARI among the three groups showed a statistically significant difference (p<0.05). The mean difference of frequency distribution of the ARI among the three groups was highly significant (p<0.001). Scanning electron micrographs of Transbond XT and Grandio revealed the uniform flow of adhesive on the molar tube base with no air bubble entrapment. Transbond Supreme LV displayed air bubbles, might be linked with less viscosity of the material, however presence of these air bubbles didn't decrease the Shear bond strength.

## Conclusion:

It is of interest to compare the bonding characteristics of the two-nanofilled adhesives, Grandio (Voco, Cuxhaven, Germany) and Transbond Supreme LV (TSLV, 3M Unitek, Monrovia, California) with conventional bonding adhesive Transbond XT (TBXT, 3M Unitek) for bonding of molar tubes. SBS of Grandio was significantly lower when compared to Transbond XT. SBS of Transbond Supreme LV was comparable to Transbond XT. SBS of Grandio was comparable to Transbond Supreme LV. Adhesive failure (between molar tube and adhesive) was observed for Transbond XT and Transbond Supreme LV whereas cohesive failure (within the adhesive itself) was observed for Grandio. Scanning Electron Microscope images revealed the uniform flow of the adhesive in the mesh network with greater porosities in Transbond Supreme LV, but it didn't seem to affect shear Bond Strength values. The results showed that nano-filled adhesive could be used as a suitable alternative for conventional adhesive but their use in clinics must need cautions. The next step for evaluating the performance of these materials would be a clinical trial.

## Figures and Tables

**Figure 3:**
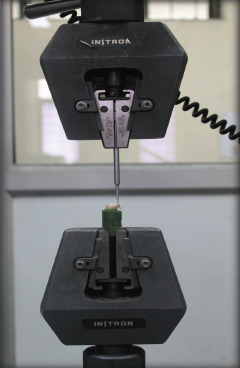
Force application in occluso-gingival direction

**Figure 4:**
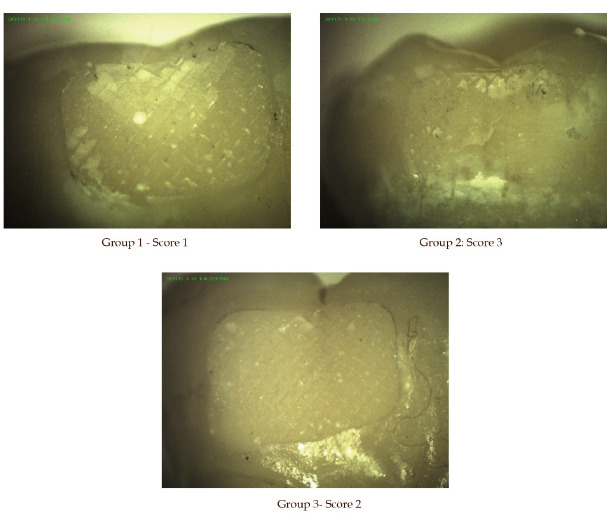
Stereomicroscope images of molar tooth surface at 20x magnification for determining Adhesive Remnant Index score
